# Supplementary material for: The catalytic activity of microRNA Argonautes plays a modest role in microRNA star strand destabilization in C. elegans
Source: bioRxiv. 2024 Jan 9:2023.01.19.524782. Originally published 2023 Jan 20. Preprint. [Version 2] doi: 10.1101/2023.01.19.524782 (PMC9882359; doi:10.1101/2023.01.19.524782)
Supplement: Supplement 1 [file media-1.pdf]

List of Supplemental Materials

Figure S1. Western blot of FLAG-tagged wild type and AEDH mutant Argonautes
Figure S2. Effect of RNAi on phenotype in AEDH mutant backgrounds
Figure S3. Spike in normalization supports minor changes in miRNA abundances
Figure S4. Validation of differential expression by qPCR
Figure S5. Standard curves for absolute miRNA quantification by qPCR
Figure S6. Polymorphism in *gpap-1* 5' UTR likely contributes to decreased expression
Figure S7. Reproducibility of miRNA changes in *ebax-1(null)* and across two *alg-1(AEDH); alg-2(AEDH)* experiments

Table S1. Strains used in this study
Table S2. Alleles generated and oligonucleotides used in this study
Table S3. Samples used for deep sequencing
Table S4. Raw miRNA and spike-in reads for all samples
Table S5. Results of small RNA sequencing analysis in embryo samples*: alg-1(AEDH); alg-2(AEDH)* versus wild type
Table S6. Results of small RNA sequencing analysis in L4 samples: *alg-1(AEDH); alg-2(AEDH)* versus wild type
Table S7. Results of small RNA sequencing analysis in adult samples: *alg-1(AEDH); alg-2(AEDH)* versus wild type (experiment 1 samples)
Table S8. Results of small RNA sequencing analysis in embryo samples: *alg-1(AEDH)* versus wild type
Table S9. Results of small RNA sequencing analysis in embryo samples: *alg-2(AEDH)* versus wild type
Table S10. Results of RNA-seq analysis in embryo samples: *alg-1(AEDH); alg-2(AEDH)* versus wild type
Table S11. Results of RNA-seq analysis in L4 samples: *alg-1(AEDH); alg-2(AEDH)* versus wild type
Table S12. Results of RNA-seq analysis in adult samples: *alg-1(AEDH); alg-2(AEDH)* versus wild type
Table S13. Results of small RNA sequencing analysis in adult samples: *ebax-1(null)* versus wild type
Table S14. Results of small RNA sequencing analysis in adult samples: *ebax-1(null); alg-1(AEDH); alg-2(AEDH) versus ebax-1(null)*
Table S15. Results of small RNA sequencing analysis in adult samples: *alg-1(AEDH); alg-2(AEDH)* versus wild type (experiment 2 samples)
Table S16. Results of small RNA sequencing analysis in adult samples: *Y47(mir-243bs_del); alg-1(AEDH); alg-2(AEDH)* versus *Y47(mir-243bs_del)*

Supplemental Figures

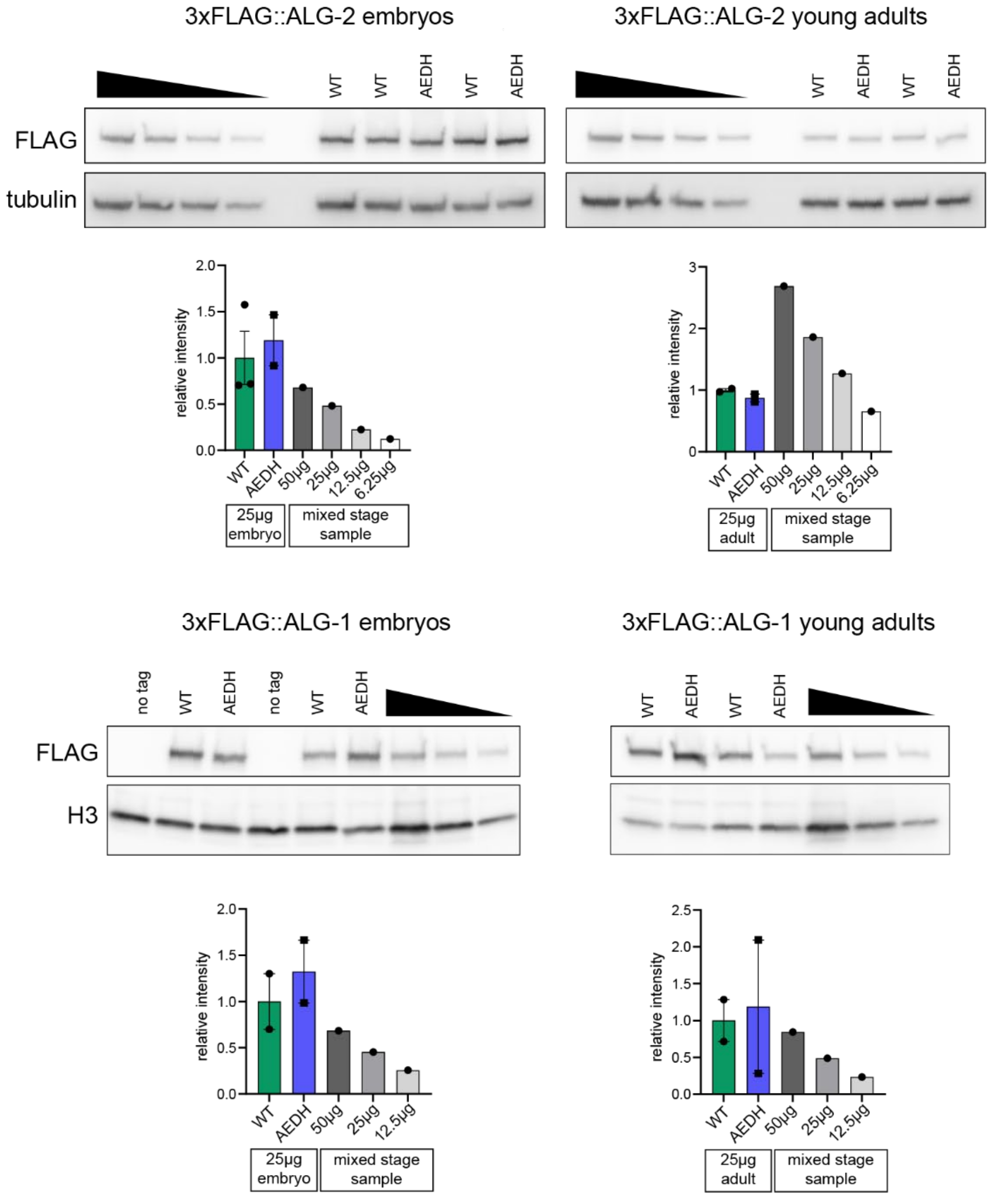

Figure S1. Western blot of FLAG-tagged wild type and AEDH mutant Argonautes. Two to three biological replicates of each genotype were quantified. For experimental samples, 25µg of protein is loaded per lane. A dilution series of a mixed-stage sample is included to calibrate fold changes. For experimental samples, FLAG signal was divided by loading control signal in the same lane to determine normalized FLAG signal; for dilution series samples, normalized FLAG signal was calculated by dividing each FLAG signal by the average of the wild type experimental sample loading control signals. Each normalized FLAG signal was then divided by the average normalized FLAG signal for all wild type samples.

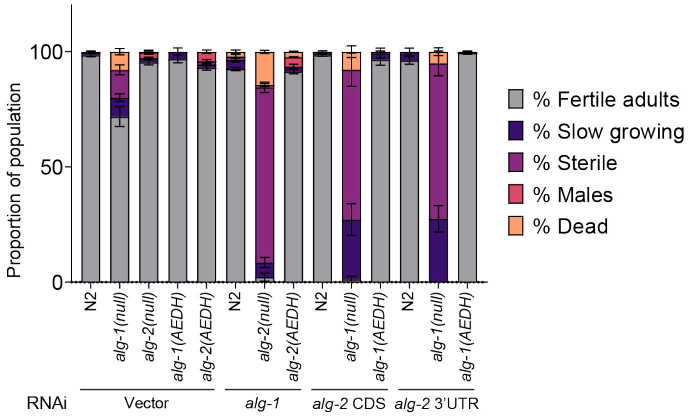

Figure S2. Effect of RNAi on phenotype in AEDH mutant backgrounds. RNAi and phenotype scoring was performed as in Bouasker, et al. Eight plates were scored per genotype/RNAi and treated as replicates. n > 470 animals per genotype/RNAi condition. Mean and SEM are plotted. *AEDH* mutations do not behave as loss-of-function alleles since they display no synthetic lethality with RNAi of the other ALG paralog.

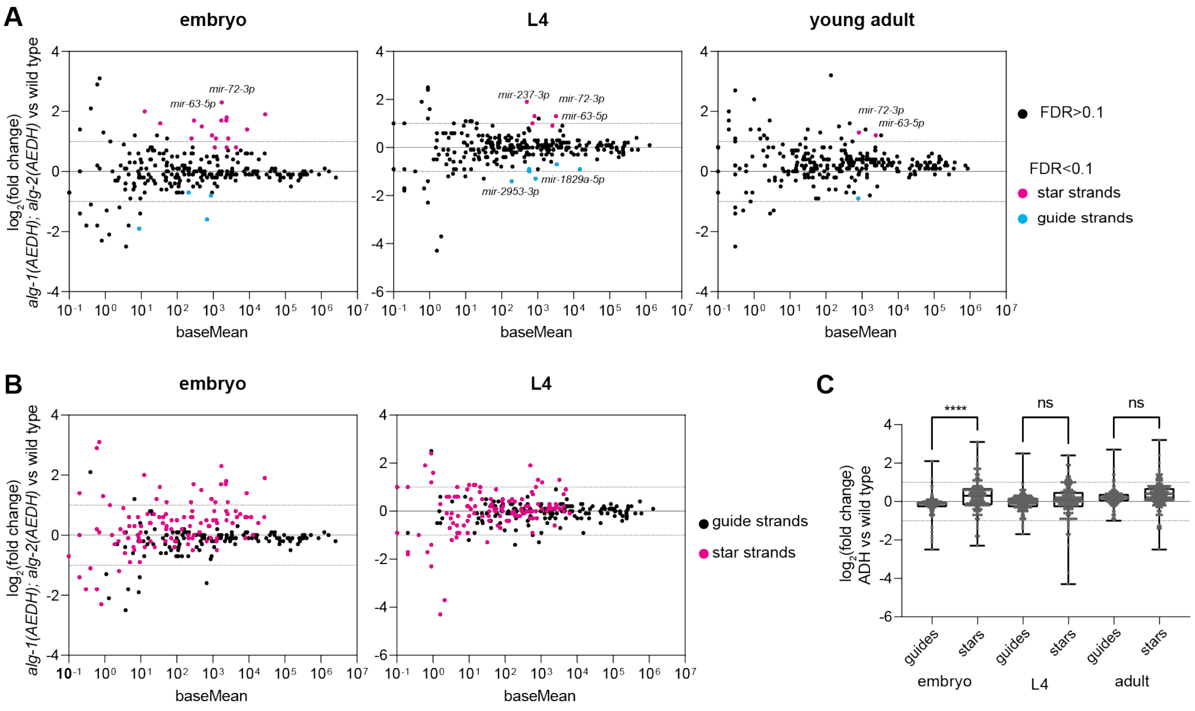

Figure S3. Spike in normalization supports minor changes in miRNA abundances. A) MA plot showing average abundance on X-axis and log$_2$(fold change) on Y-axis. miRNAs showing significant changes in *alg-1(AEDH); alg-2(AEDH)* versus wild type are shown in blue (miRNA guide strands) or pink (star strands) (DEseq2 FDR<0.1). Reads were normalized to exogenous spike-ins (See methods and Table S2). Three (L4) or four (embryo and young adult) biological replicates of each genotype were analyzed. (B) MA plot as in (A), with all guide strands shown in black and all star strands in pink. (C) Summary of log$_2$(fold change) values for all guides or star strands in each stage in *alg-1(AEDH); alg-2(AEDH)* versus wild type. Log$_2$(fold change) of star strands were compared to those of guide strands in the same sample by one-way ANOVA followed by Sidak's multiple comparisons test. ***p<0.0001

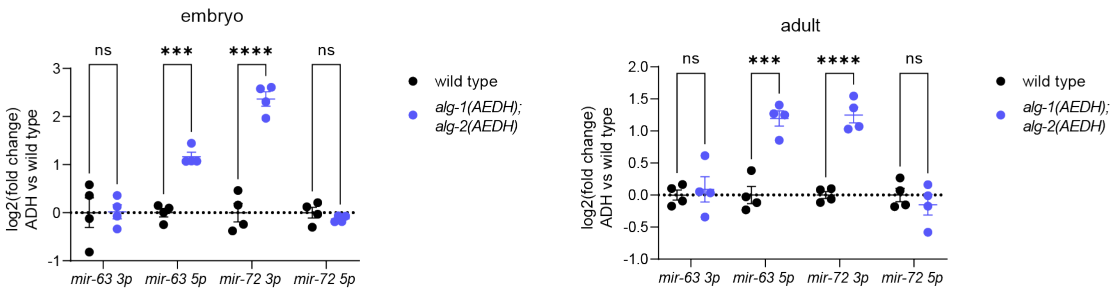

Figure S4. Validation of differential expression by qPCR. miRNA Taqman-qRT-PCR assays validate increased levels of *mir-63-5p* and *mir-72-3p* in the *alg-1(AEDH); alg-2(AEDH)* background. Unpaired t-tests, ****p-value < 0.0001, ***p-value < 0.001.

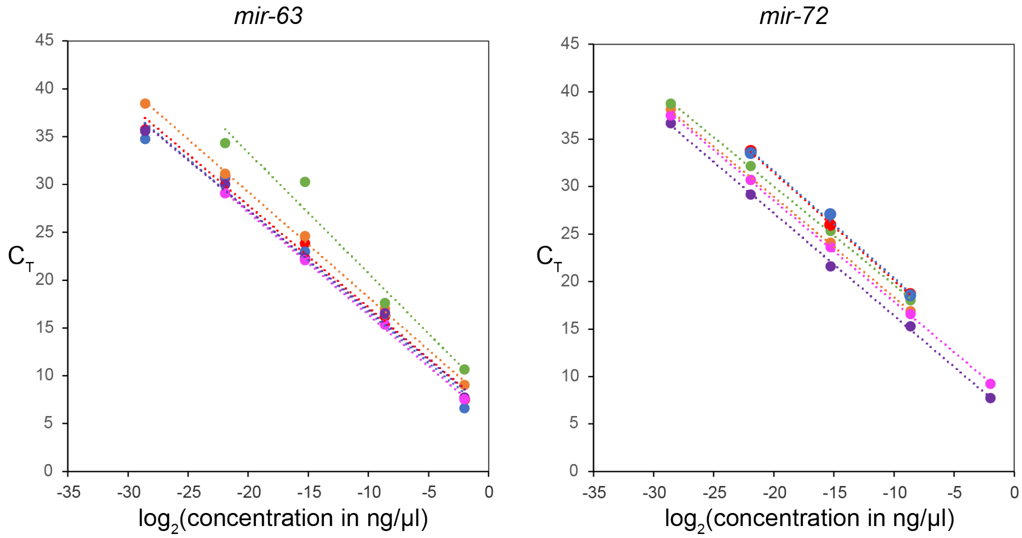

mir-63-3p	y = -1.0665x + 6.4959	R² = 0.9907
mir-63-5p	y = -1.0633x + 6.0536	R² = 0.9816
mir-63-3p_mut2	y = -1.0993x + 7.2333	R² = 0.9988
mir-63-5p_mut2	y = -1.0436x + 6.4644	R² = 0.9944
mir-63-3p_mut3	y = -1.074x + 5.6509	R² = 0.9990
mir-63-5p_mut3	y = -1.26x + 8.1393	R² = 0.9660

mir-72-3p	y = -1.1342x + 8.7734	R² = 0.9998
mir-72-5p	y = -1.1278x + 9.12	R² = 0.9933
mir-72-3p_mut1	y = -1.0592x + 7.7451	R² = 0.9996
mir-72-5p_mut1	y = -1.0799x + 5.5879	R² = 0.9992
mir-72-3p_mut2	y = -1.0636x + 7.2479	R² = 0.9998
mir-72-5p_mut2	y = -1.0377x + 9.2635	R² = 0.9995

Figure S5. Standard curves for absolute miRNA quantification by qPCR. Synthetic RNA oligonucleotides were diluted to indicated concentrations, and 1.66µl was used in 5µl RT reactions and downstream miRNA-Taqman as indicated in methods. Average of three technical replicates is shown. Highest and lowest concentrations were excluded from the curve if they were outside the linear range. All experimental sample concentrations fell within the linear range of the respective assay.

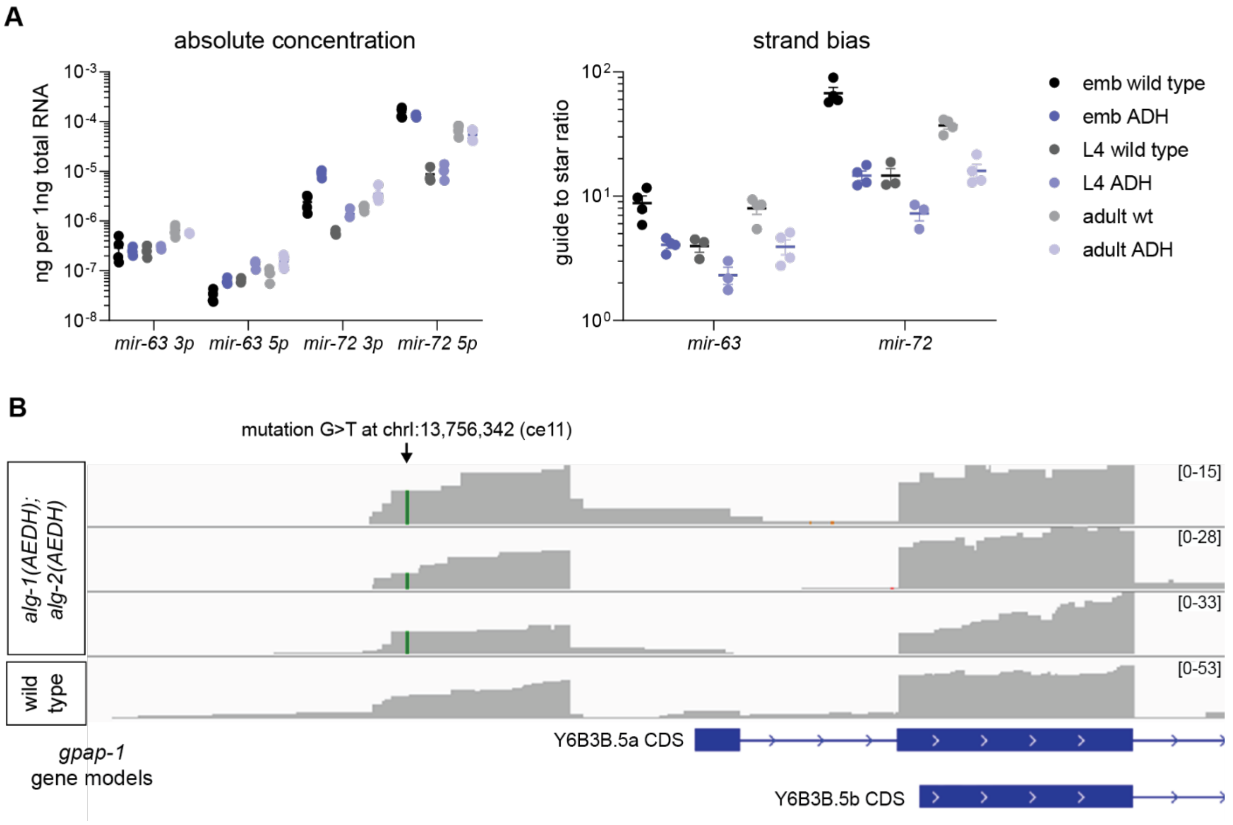

Figure S6. Polymorphism in *gpap-1* 5' UTR likely contributes to decreased expression. A) Left: Absolute quantification by qPCR of *mir-63* and *mir-72* guide and star strands Right: Strand bias calculated from absolute quantification by qPCR. Mean and SEM of three to four biological replicates per condition are shown. B) One gene was identified as mis-regulated across more than one developmental stage in the *alg-1(AEDH);alg-2(AEDH)* mutant strain. Close examination of RNA-seq data revealed that this gene, *gpap-1*, contains a polymorphism in its 5' UTR in the mutant strain.

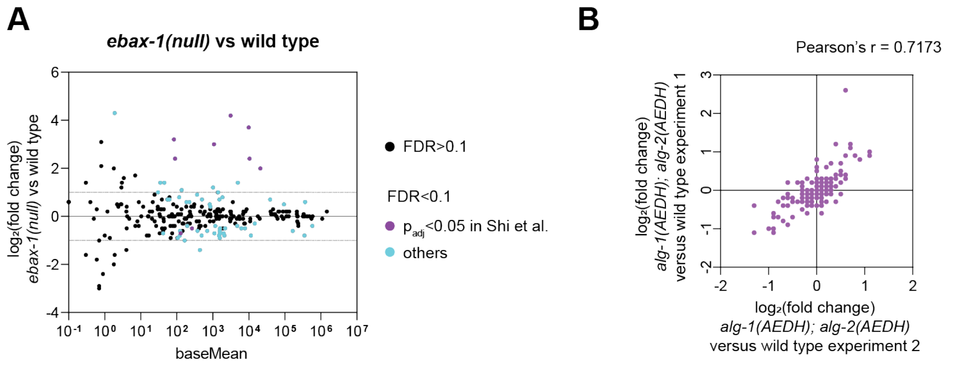

Figure S7. Reproducibility of miRNA changes in *ebax-1(null)* and across two *alg-1(AEDH); alg-2(AEDH)* experiments. A) MA plots showing average abundance on X-axis and $\log_2$(fold change) on Y-axis. miRNAs showing significant changes are highlighted in light blue or purple (DEseq2 FDR<0.1); upregulated miRNAs that were previously noted in Shi, et al are in purple. Four biological replicates of each genotype were analyzed. B) Correlation of changes induced by *alg-1(AEDH); alg-2(AEDH)* in wild type experiment 1 (X-axis) or experiment 2 (Y-axis). Each experiment consists of four biological replicates of each genotype. Only miRNAs with baseMean > 50 in wild type experiment 1 are included. (A-B) All samples are from young adults.
